# Biosurfactants as Anticancer Agents: Glycolipids Affect Skin Cells in a Differential Manner Dependent on Chemical Structure

**DOI:** 10.3390/pharmaceutics14020360

**Published:** 2022-02-04

**Authors:** Simms A. Adu, Matthew S. Twigg, Patrick J. Naughton, Roger Marchant, Ibrahim M. Banat

**Affiliations:** 1 The Nutrition Innovation Centre for Food and Health (NICHE), Faculty of Life and Health Sciences, School of Biomedical Sciences, Ulster University, Coleraine BT52 1SA, UK; adu-s@ulster.ac.uk (S.A.A.); pj.naughton@ulster.ac.uk (P.J.N.); 2 Pharmaceutical Science Research Group, Biomedical Science Research Institute, Ulster University, Coleraine BT52 1SA, UK; m.twigg@ulster.ac.uk (M.S.T.); roger.marchant@ulster.ac.uk (R.M.)

**Keywords:** biosurfactant, glycolipid, anticancer, melanoma, microbiology

## Abstract

Melanomas account for 80% of skin cancer deaths. Due to the strong relationship between melanomas and U.V. radiation, sunscreens have been recommended for use as a primary preventative measure. However, there is a need for targeted, less invasive treatment strategies. Glycolipids such as sophorolipids and rhamnolipids are microbially derived biosurfactants possessing bioactive properties such as antimicrobial, immunomodulatory and anticancer effects. This study aimed to ascertain the differing effects of glycolipids on skin cells. Highly purified and fully characterized preparations of sophorolipids and rhamnolipids were used to treat spontaneously transformed human keratinocyte (HaCaT) and the human malignant melanocyte (SK-MEL-28) cell lines. Cell viability and morphological analyses revealed that glycolipids have differential effects on the skin cells dependent on their chemical structure. Lactonic sophorolipids and mono-rhamnolipids were shown to have a significantly detrimental effect on melanoma cell viability compared to healthy human keratinocytes. These glycolipids were shown to induce cell death via necrosis. Additionally, sophorolipids were shown to significantly inhibit SK-MEL-28 cell migration. These findings suggest that glycolipids could be used as bioactive agents with selective inhibitory effects. As such, glycolipids could be a substitute for synthetically derived surfactants in sunscreens to provide additional benefit and have the potential as novel anti-skin-cancer therapies.

## 1. Introduction

A malignancy of melanin-producing cells in the epidermis (melanocytes) is referred to as a melanoma. Due to these cells producing a variety of different signaling factors that promote migration, metastasis of melanomas is a major characteristic of this form of cancer. Although melanomas only represent 1% of all skin cancers, they account for 80% of skin-cancer-related deaths [1]. According to the World Health Organization’s Global Cancer Observatory (GLOBOCAN), there were 324,635 newly reported melanoma cases in 2020, which represented 1.7% of global cancer diagnoses for that year [2]. In the past 50 years, melanoma incidence has seen an increasing rise in developed countries (320% increase in the U.S. since 1975), with a strong prevalence towards affecting fair-skinned persons [3]. As melanomas are mainly caused by increased U.V. light exposure, and with climate change forecasts predicting increased sun exposure in Northern Europe and North America in the coming decades, this trend is expected to continue. As with many forms of cancer, treatment of melanoma is dependent on the stage of the cancerous tissue. At stage 1 and 2 treatment is primarily via surgical excision of the melanoma and surrounding skin tissue [4]. Treatment of stage 3 and 4 melanomas (where the cancer has spread to the lymph nodes and further to other organs) is via surgical removal of the affected lymph tissue followed by non-targeted chemotherapy and/or radio therapy [4]. Several immunotherapy options have been developed for melanoma treatment at stage 3–4. These include, but are not limited to, treatment with interferon α2b, interleukin-2, anti-CTLA-4 antibodies (Ipilimumab) and anti-programmed cell death protein 1 antibodies (Nivolumab) [5,6,7,8]. However, many of these treatments are non-specific, affecting both healthy and cancer-driven immunological responses, and can be very costly to the healthcare provider [4]. It is therefore commonly accepted that due to the significantly unpleasant side effects to the patient associated with these treatments, prevention or targeted treatment of melanoma is a preferable alternative option. The principal way of preventing melanoma is by stopping U.V. radiation from reaching the skin with protective sunscreen formulations. Currently these formulations do not possess ingredients that can also specifically target melanoma cells for destruction. However, they do contain synthetically produced surfactant compounds such as sodium lauryl ether sulphate (SLES). Replacing these synthetic surfactants with alternative, naturally produced compounds that have antimelanoma activity would provide added functionality to these formulations. Such substitutes are microbially produced biosurfactants, sustainably produced compounds that have similar physical action and potential anticancer activity.

Biosurfactants are secondary metabolite compounds produced by microorganisms such as bacteria and fungi which affect surface chemistry, lowering surface interphase tension and aiding in the formation of emulsions, gels and foams [9]. Biosurfactants are categorized by their molecular structure, with one of the most extensively researched groups being glycolipids [10]. Glycolipids comprise a hydrophilic mono or disaccharide moiety joined via a covalent linkage to a hydrophobic saturated or unsaturated fatty acid comprising between 8–18 carbons [11]. Glycolipids can be further classified based on their sugar moiety. Two of the most abundant groups of microbially synthesized glycolipids are the rhamnolipids produced by bacteria such as *Pseudomonas aeruginosa* and *Burkholderia thailandensis*, and sophorolipids which are produced by yeasts such as *Starmerella bombicola* [12,13,14]. As glycolipid biosurfactants have significantly reduced toxicity and increased biocompatibility compared to petrochemically derived synthetic surfactant compounds, their potential for commercial exploitation is extensive and includes biomedical application such as skin care pharmaceutical formulations [15,16]. Additionally, glycolipid biosurfactants, some of which are produced by microbes isolated from environmental sources such as the open ocean, have been investigated for their ability to affect tumour progression and therefore act as anticancer agents [17,18].

In vitro, sophorolipids have been shown to have cytotoxic effects against human pancreatic (HPAC), liver (H7402), lung (A549), brain (LN229, HNCG-2), oesophageal (KYSE109, KYSE450), breast, cervical (HeLa), leukemic (HL60, K562) and colonic (HCT116, CaCo-2) cell lines [19,20,21,22,23,24,25,26]. Rhamnolipid biosurfactants have also been shown to have anticancer effects against breast cancer (MCF-7), colon cancer (CaCo-2), liver cancer (HepG2) and human promyelocytic leukaemia cell lines [27,28]. There has been little investigation of the effects biosurfactants such as glycolipids have on melanoma; however, Haque et al., 2021 have shown that glycolipids negatively affected the viability of murine melanoma cell line B16F10 [29]. Many of the previous studies that have investigated the anticancer effects of glycolipid biosurfactants utilized uncharacterized mixtures of different glycolipids or only focused on a single class of glycolipid. In this study, we determined the differential effect preparations of rhamnolipids and sophorolipids had on melanoma cells specific to their chemical structures compared to their effect on healthy skin cells. Additionally, unlike previous studies the glycolipid preparation utilized in this study were highly purified and fully characterized via chemical analysis prior to in vitro testing. Finally, we assessed the effects of rhamnolipids and sophorolipids on in vitro wound healing and inhibition of cancer cells migration. We showed that with the in vitro skin cell lines tested, anticancer activity appears to be highly related to chemical structure with glycolipids possessing small differences in structure, producing observably differential effects on the cell lines tested.

## 2. Materials and Methods

### 2.1. Purification of Glycolipids

Purified diacetylated lactonic and non-acetylated acidic sophorolipid preparations utilized in this study were purchased separately from *Biosynth Carbosynth*, Compton, UK (DL71483 and DL71484 respectively). Mono-rhamnolipid and di-rhamnolipid preparations used in this study were purified in house from a crude mixture of rhamnolipid congeners obtained from *Daqing Victex Chemical Co., Ltd.*, Daqing, China. Purification was achieved in a two-step process. Initially, liquid phase extraction was performed three times in 0.5% vol. HPLC Grade Ethyl Acetate (*Merck*, Gillingham, UK). The solvent fraction was retained and dried via rotary evaporation at 50 °C under vacuum. The extract was then reconstituted in HPLC Grade Chloroform (*Merck*, Gillingham, UK) and subjected to solid phase extraction using Strata SI-1 Silica (55 μm, 70 Å) Giga tubes (*Phenomenex*, Macclesfield, UK) which had been preconditioned by washing with HPLC Grade Chloroform (*Merck*, Gillingham, UK). Mono-rhamnolipid and di-rhamnolipid congeners were eluted from the column separately with HPLC Grade Chloroform: Methanol (*Merck*, Gillingham, UK) at 5:0.5 (*v*/*v*) and 5:5 (*v*/*v*) respectively [30]. All glycolipid preparation were resuspended in HPLC Grade methanol at a stock concentration of 1 mg mL^−1^ and stored at −20 °C. Experimental concentrations of each glycolipid preparation were generated by diluting from the stock concentration using HPLC Grade Methanol (*Merck*, Gillingham, UK).

### 2.2. Characterization of Purified Glycolipids Preparations

All glycolipid preparations utilized in this study were fully characterized for congener composition. This characterization was achieved using High Performance Liquid Chromatography—Mass Spectrometry (HPLC-MS). MS analysis was carried out using a LCQ Classic electrospray ion-trap spectrometer (*Thermo Scientific*) as described by Smyth et al., 2010. Each glycolipid preparation was dissolved in HPLC grade methanol (*Merck*, Gillingham, UK) at a concentration of 1 mg mL^−1^. The conditions of operation for the HPLC-MS were set as follows: Mobile phase A for the LC device was Elga HPLC grade water (*Merck*, Gillingham, UK) whereas mobile Phase B was Acetonitrile (*Merck*, Gillingham, UK). The ambient temperature inside the column was sufficient for operation. The flow rate was set at 0.8 mL/min with injection volume of 5 µL and UV detection at 220 nm. The system was coupled with Electrospray ionization mass spectrometer (ESI/MS), set in negative mode. Nitrogen for sheath and auxiliary gas was set with arbitrary values of 70 and 8, respectively. Spray voltage was set at 4.5 kV and capillary temperature at 250 °C. Scanning range of masses for all biosurfactants was set at 300–1000 *m*/*z* with a maximum runtime of approximately 40 min [30].

### 2.3. Cell Culture

The cell lines utilized in this study were an in vitro spontaneously transformed human keratinocyte HaCaT, obtained from *AddexBio*, San Diego, CA, USA (T0020001/117), and the human malignant melanocyte SK-MEL-28 (ATCC HTB-72), kindly gifted by Dr. Paul Thomson (Ulster University, UK). HaCaT cells were routinely cultured in Dulbecco’s Modified Eagle medium (DMEM) high glucose (*ThermoFisher Scientific*, Loughborough, UK) supplemented with 10% (***v*/*v***) foetal bovine serum (FBS) (*ThermoFisher Scientific*, Loughborough, UK). SK-MEL-28 were routinely cultured in RPMI medium 1640 (*ThermoFisher Scientific*) supplemented with 10% (***v*/*v***) FBS (*ThermoFisher Scientific*, Loughborough, UK). All cell culture took place at 37 °C in a humidified atmosphere containing 5% CO_2_.

### 2.4. Cell Viability Assays

Cell viability following treatment with glycolipids was assessed by means of a cell proliferation assay II (XTT) (*Roche*, Welwyn Garden City, UK). HaCaT and SK-MEL-28 were cultured to confluency in 10 mL of complete media within a T75 flask. Cells were then seeded into a 96-well cell culture plate (*Sarstedt*, Leicester, UK) at 1 × 10^4^ cells per well and allowed to attached overnight. The medium was then aspirated, and the cells cultured in 100 μL per well serum-free media for 24 h. The serum-free media was then aspirated, and the cells were cultured for a further 24 h in either complete medium, complete medium supplemented with incremental concentrations of each glycolipid preparation, complete medium supplemented with 100 µg mL^−1^ synthetic surfactants SLES (*R & D Laboratories Limited*, Antrim, UK) or complete medium supplemented with 1% (***v*/*v***) HPLC grade methanol (vehicle control) (all at 100 μL per well). Following treatment, media were aspirated and the cells washed with sterile phosphate buffered saline (PBS) (*ThermoFisher Scientific*, Loughborough, UK). Fifty microliters per well of XTT reagent were then added to the cells and incubated for 4 h. Following incubation, absorbance was measured at 450 and 650 nm. Cell viability experiments were carried out independently three times with six replicates per treatment group.

### 2.5. Cell Morphology Assessment

Cell morphology following treatment with glycolipids was assessed using visible light microscopy. HaCaT and SK-MEL-28 were cultured to confluency in 10 mL of complete medium within a T75 flask. Cells were then seeded into a 12-well cell culture plate (*Sarstedt*, Leicester, UK) at 1 × 10^5^ cells per well and allowed to attached overnight. The medium was then aspirated, and the cells were cultured in 1 mL per well of serum-free medium for 24 h. The serum-free medium was aspirated and the cells were cultured for a further 24 h in either complete medium; complete medium supplemented with 100 μg mL^−1^ of each glycolipid preparation; complete medium supplemented with 100 µg mL^−1^ synthetic surfactants SLES (*R & D Laboratories Limited*, Antrim, UK) or complete medium supplemented with 1% (*v*/*v*) HPLC grade methanol (vehicle control) (all at 1 mL per well). For acidic sophorolipids and mono-rhamnolipids, a further experiment increasing treatment concentration to 500 μg mL^−1^ was also carried out. Following treatment, the morphology of each cell line was assessed by directly imaging the cells in the wells at 200× magnification using a Digital Sight DS-L1 camera (*Nikon Europe B. V.*, Amsterdam, The Netherlands) attached to an Eclipse TS100 inverted microscopy (*Nikon Europe B. V.*, Amsterdam, The Netherlands). Cell morphology observations were carried out independently three times with three replicates per treatment group. Each replicate was imaged in three independent locations within the well and a representative image was selected at random (by computer) for publication.

### 2.6. Acridine Orange and Propidium Iodine Staining

To assess the distinct morphological pattern of cell death induced by Glycolipids in HaCaT and SK-MEL-28 melanoma cell line, the cells were stained with acridine orange (AO) and propidium iodide (PI) (*Merck*, Gillingham, UK) [31]. HaCaT and SK-MEL-28 cells were cultured, seeded onto Nunc 12-well cell culture plates and treated with glycolipid and synthetic SLES preparations as previously described in Section 2.4. Following glycolipid treatment, the cells were washed three times with PBS (*ThermoFisher Scientific*, Loughborough, UK) to remove floating cells and subsequently incubated with AO and PI for 3 min, each prepared at a working concentration of 100 ug mL^−1^ and mixed at a ratio of 1:1. Cells were then rewashed three times with PBS (*ThermoFisher Scientific*, Loughborough, UK) and stained cells were imaged at 200× magnification using and Eclipse TS100 fluorescence microscope (*Nikon Europe B. V.*, Amsterdam, The Netherlands). The excitation and emission wavelengths for AO were 493 and 535 nm, and for PI they were 535 and 614 nm. Three images per well were randomly selected (by computer) and processed with ImageJ Software for each of the three independent experimental replicates [32].

### 2.7. Scratch Assay

For in vitro wound healing and cell migration assay, HaCaT and SK-MEL-28 melanoma cell lines were seeded at 3 × 10^5^ cells per well in 6-well cell culture plates (*Sarstedt,* Leicester, UK) and allowed to attach overnight. Cells were then serum starved for 24 h. Post serum-starvation, spent medium was replaced with sterile prewarmed PBS (*ThermoFisher Scientific*, Loughborough, UK). The confluent monolayer of cells was uniformly scraped vertically across wells using sterile 200 µL pipette tips. To remove cell debris and cells accumulated on the scratch surface, the plates were washed three times with sterile prewarmed PBS (*ThermoFisher Scientific*, Loughborough, UK). Cells were then treated with complete media containing 1% (***v*/*v***) methanol (*Merck*, Gillingham, UK) (vehicle control), each glycolipid preparation and SLES (both at 20 μg mL^−1^). Cell migration was evaluated at 0 and 24 h using a Digital Sight DS-L1 camera (*Nikon Europe B. V.*, Amsterdam, The Netherlands). attached to an Eclipse TS100 inverted microscope (*Nikon Europe B. V.*, Amsterdam, The Netherlands) at (×100 magnification). The total area of the scratch, and area of the scratch remaining uncovered by cells following 24 h treatment, was calculated from a random selection of the images taken for each treatment group using ImageJ Software [32,33]. The percentage of the scratch uncovered by cells for each treatment was then calculated and plotted. The experiment was repeated three independent times.

### 2.8. Statistical Analysis

Statistical analyses of all data were carried out using Prism v5.0 for Windows (*GraphPad Software*, San Diego, CA, USA). Cell viability and scratch assay data were analyzed via a two-way ANOVA followed by Bonferroni post hoc testing. The significance of the results was tested at *p* < 0.05 level. IC_50_ values were determined from three independently carried out cell viability assays and statistical significance determined via unpaired *t*-test; results were tested at *p* < 0.05 level for each treatment group.

## 3. Results

### 3.1. Characterization of Glycolipid Preparations

Sophorolipids utilized in this study were purchased from a commercial source as separate preparations consisting of primarily non-acetylated acidic sophorolipid congeners and diacetylated lactonic sophorolipid congeners. Using HPLC-MS analysis, the congener composition and percentage relative abundance of each of these preparations was identified (Table 1). This analysis showed that the non-acetylated acidic sophorolipid preparation was 100% pure, and that the diacetylated lactonic sophorolipid preparation was 89.86% pure. The dominant congeners in each preparation were identified as acidic-SL C18:1 (65.53%) and lactonic SL, R1 + R2 = Ac, C18:1 (63.40%), respectively. The rhamnolipids utilized in this study were purchased as crude extraction from a commercial source. This crude extract was then purified and separated into preparations of mono-rhamnolipid congeners and di-rhamnolipids congeners. As with the sophorolipid preparations, the mono and di-rhamnolipids preparations were analyzed for purity and congener composition via HPLC-MS (Table 1). The mono-rhamnolipid preparation was shown to be 96% pure while the di-rhamnolipid preparation was 97% pure. The dominant congeners in each preparation were identified as Rha-C_10_-C_10_ (84.40%) and Rha-Rha-C_10_-C_10_ (57.99%), respectively. For HPLC-MS profiles of each preparation see Figure S1 (Supplementary material).

### 3.2. Glycolipids Affect the Viability of HaCaT and SK-Mel-28 Cell Differentially According to Molecular Structure

**Figure 1:**
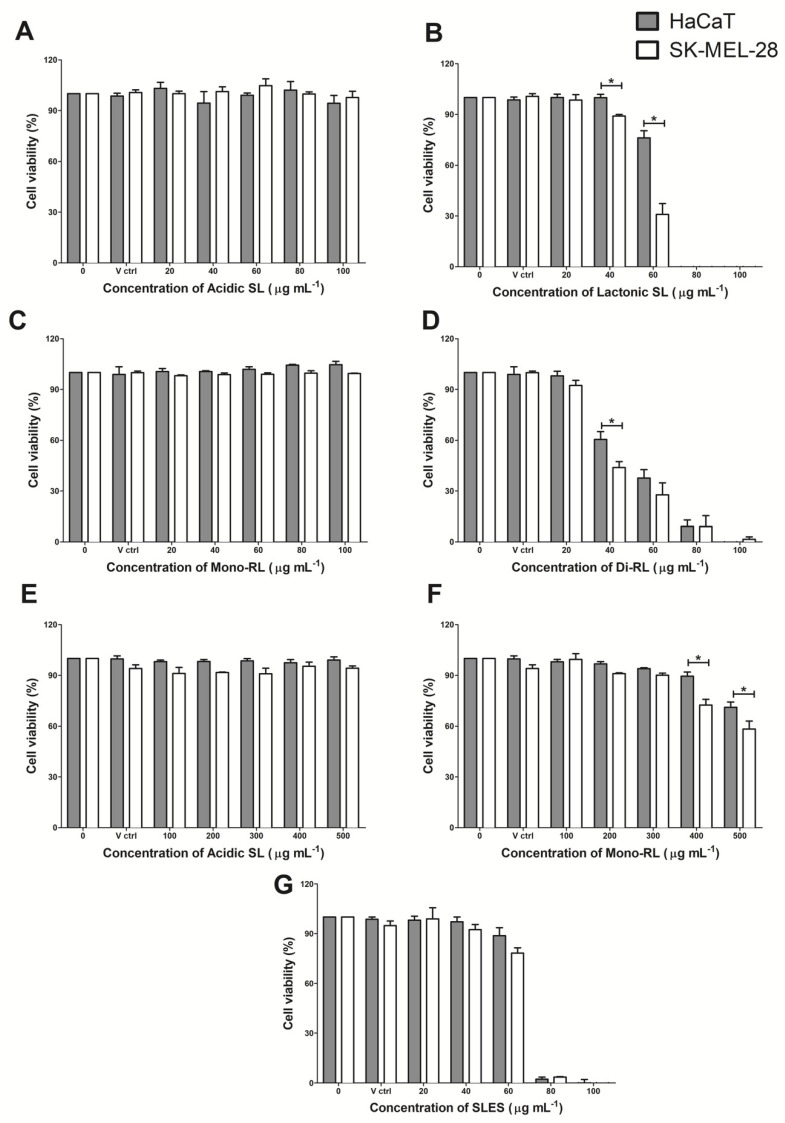
The effect on HaCaT and SK-MEL-28 cell viability when treated with (**A**) acidic sophorolipids at 0–100 μg mL^−1^; (**B**) lactonic sophorolipids at 0–100 μg mL^−1^; (**C**) mono-rhamnolipids at 0–100 μg mL^−1^; (**D**) di-rhamnolipids at 0–100 μg mL^−1^; (**E**) acidic sophorolipids at 0–500 μg mL^−1^; (**F**) mono-rhamnolipids at 0–500 μg mL^−1^; (**G**) SLES at 0–100 μg mL^−1^. Data are the mean results of three independently conducted experiments, each with six replicates per treatment group. Error bars show standard error from the mean. Statistical significance was determined using a two-way ANOVA followed by Bonferroni pos hoc test, * = *p* ≤ 0.05.

Human keratinocyte and malignant melanocyte cell lines HaCaT and SK-Mel-28 were initially treated with acidic sophorolipid, lactonic sophorolipid, mono-rhamnolipid and di-rhamnolipid preparations at concentrations ranging from 0–100 μg mL^−1^. The cytotoxic effect of these glycolipids on the cell lines was assessed and compared to cells treated with 100 μg mL^−1^ Methanol (vehicle control). The cytotoxic effect of the synthetic surfactant compound SLES (0–100 μg mL^−1^) on both cell lines was also observed. At these concentrations acidic sophorolipids and mono-rhamnolipids had no significant cytotoxic effect on either cell line (Figure 1). Lactonic sophorolipids significantly reduced (*p* ≤ 0.05) the viability of SK-MEL-28 cell at concentrations above 40 μg mL^−1^ and HaCaT above 60 μg mL^−1^, with a significantly detrimental effect on the SK-MEL-28 cell line compared to HaCaT (40 μg mL^−1^ *p* ≤ 0.05; 60 μg mL^−1^ *p* ≤ 0.001). Di-rhamnolipids significantly (*p* ≤ 0.05) reduced the viability of both cell lines at concentrations above 40 μg mL^−1^, with a significantly detrimental effect on the SK-MEL-28 cell line compared to HaCaT at 40 μg mL^−1^ (*p* ≤ 0.05). Both cell lines treated with SLES had significantly (*p* ≤ 0.05) reduced viabilities at treatments over 60 μg mL^−1^ but with no difference between each cell line (Figure 1). The IC_50_ value for lactonic sophorolipid was significantly lower for the SK-Mel-28 melanocyte cell lines than it was for the HaCaT cell line (Table 2). This indicates that significantly less lactonic sophorolipid is required to have a detrimental effect on malignant melanocytes than on healthy skin cells. This trend was also observed for di-rhamnolipids but was not significant (Table 2). As no effect at concentrations up to 100 μg mL^−1^ was observed with acidic sophorolipids and mono-rhamnolipids on both cell lines, cell viability assays were repeated at concentrations up to 500 μg mL^−1^. Again, acidic sophorolipids had no significant effect on cell viability. However mono-rhamnolipids did affect the viability of each cell line at concentrations exceeding 400 μg mL^−1^ with a significantly more detrimental effect on the SK-MEL-28 cells (400 μg mL^−1^ *p* ≤ 0.001; 500 μg mL^−1^ *p* ≤ 0.01) (Figure 1). The concentration of mono-rhamnolipids required to reach an IC_50_ value was lower for the SK-Mel-28 melanocyte cell lines than it was for the HaCaT cell line; however, this was not significant (Table 2).

### 3.3. Glycolipids Affect Cell Morphology of HaCaT and SK-Mel-28 Cell Lines

**Figure 2:**
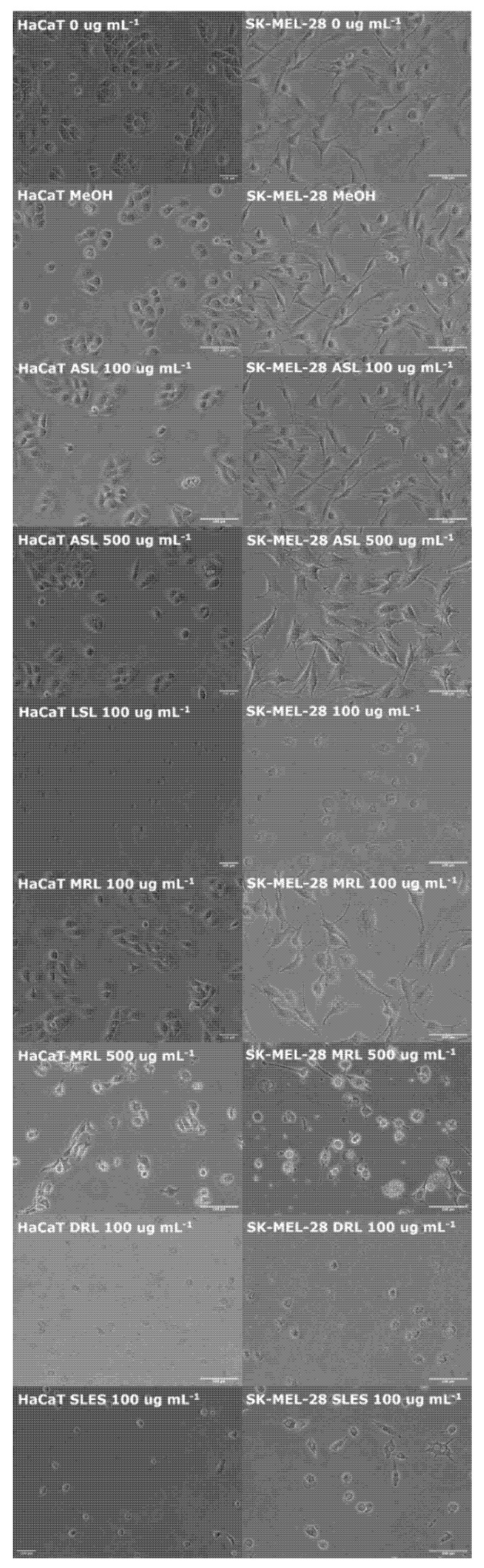
Light microscopy images of HaCaT and SK-MEL-28 cells untreated (0 μg mL^−1^) or following 24 h of treatment with a vehicle control (MeOH), acidic sophorolipid (ASL) (100 μg mL^−1^ and 500 μg mL^−1^), lactonic sophorolipid (LSL) (100 μg mL^−1^), mono-rhamnolipid (MRL) (100 μg mL^−1^ and 500 μg mL^−1^), di-rhamnolipid (DRL) (100 μg mL^−1^) and SLES at (100 μg mL^−1^). Images were randomly selected from three independently conducted experiments with each treatment group imaged three times; scale bar is 100 μm.

As cell viability assays showed that glycolipids had cytotoxic effects upon HaCaT and SK-Mel-28 cell lines, the morphology of treated cells were directly observed under light microscopy. Untreated and vehicle control treated HaCaT and SK-Mel-28 were observed to maintain a normal adherent monolayer with no unexpected morphological changes (Figure 2). Treatment with acidic sophorolipids and mono-rhamnolipids at 100 μg mL^−1^ also had no observable effect upon each cell lines morphology after 24 h (Figure 2). However, treatment at 500 μg mL^−1^ mono-rhamnolipid caused both HaCaT and SK-Mel-28 cells to become detached from the surface of the 12-well plate, taking on an abnormal circularized appearance after 24 h (Figure 2). Treatment with acidic sophorolipids at this concentration had no visible effect. Treatment with lactonic sophorolipids, di-rhamnolipids and SLES at a concentration of 100 μg mL^−1^ caused both cell lines to become detached from the 12-well plate and adopt an abnormal circularized appearance after 24 h (Figure 2).

### 3.4. Glycolipids Mediate Cell Death of HaCaT and SK-Mel-28 Cell Lines via Necrosis

**Figure 3:**
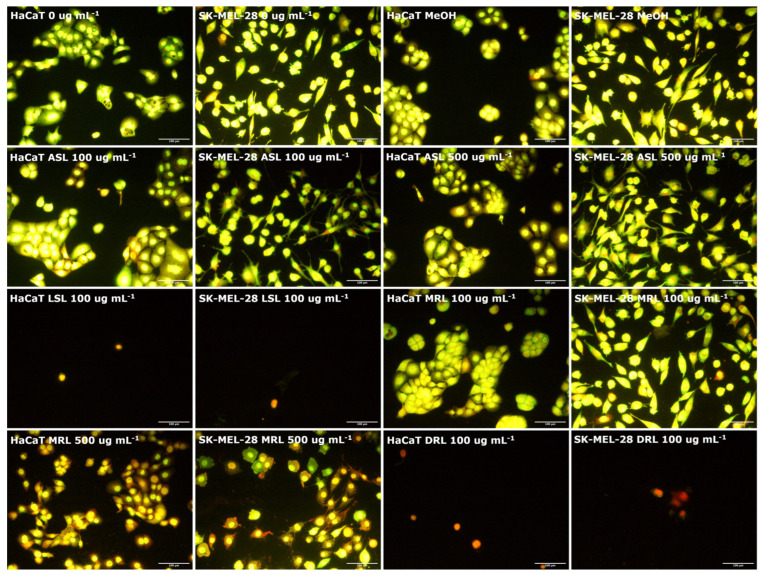
Morphological assessment of the pattern of cell death induced by glycolipids using AO/PI staining following a 24 h treatment under the same conditions as reported in Figure 2. The vast number of HaCaT and SK-MEL-28 cells treated with vehicle control, acidic sophorolipids (100 μg mL^−1^ and 500 μg mL^−1^) and 100 μg mL^−1^ of mono-rhamnolipids were morphologically viable (green stains with uncondensed nuclei) while 100 μg mL^−1^ treatment concentrations of lactonic sophorolipids, di-rhamnolipids and 500 μg mL^−1^ of mono-rhamnolipids resulted in significant reduction in the cell population, with the few adherent cells staining red/orange (necrotic cell death). Scale bar set at 100 μm.

Dual AO/PI staining is a technique for morphologically assessing necrosis or apoptosis-associated changes in the membrane and nuclei of cells after exposure to a source of injury. Acridine orange is membrane permeable and stains cell nuclei green. Propidium iodide is membrane impermeable and will only stain cell nuclei with compromised membrane integrity as red or orange. Thus, while green condensed and fragmented chromatin are the morphological hallmarks for apoptotic cells, necrotic cells retain red/orange coloration with nuclei morphology similar to that of viable cells (no condensed chromatin), but disrupted cell membrane integrity [34]. Here, we used a fluorescence microscope to morphologically assess the pattern of cell death induced by glycolipids in HaCaT and SK-MEL-28 melanoma cell line. We observed that there were no significant differences in cell population and morphology of both HaCaT and SK-MEL-28 melanoma cell line between the untreated control (0 μg mL^−^^1^), vehicle control and up to 100 μg mL^−1^ treated concentrations of acidic sophorolipids and mono-rhamnolipids (Figure 3). However, lactonic sophorolipids and di-rhamnolipids treatments up to 100 μg mL^−1^ concentrations resulted in a significant reduction in cell population for both HaCaT and SK-MEL-28 melanoma cell line with red/orange granular nuclei stains detected in the very few remaining adherent cells, which had their membranes disrupted (necrotic cell death) (Figure 3). Moreover, increasing the concentration of mono-rhamnolipids up to 500 μg mL^−1^ resulted in a similar trend but with a much higher cell population, whereas cells treatment with acidic sophorolipids at the same concentrations remained unaffected (Figure 3). Treatment with SLES resulted in necrotic cell death in both cell lines at a concentration of 100 μg mL^−1^, highly similar to that which was observed for cells treated with lactonic sophorolipids (Supplementary Figure S2).

### 3.5. Glycolipids Inhibit Cellular Migration in SK-MEL-28 Cell Lines

**Figure 4:**
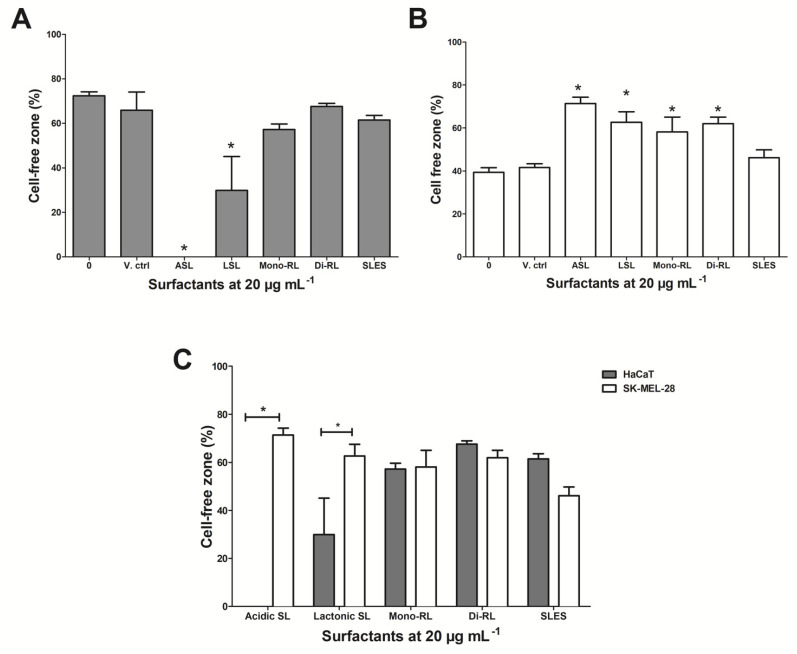
Scratch assay showing the effect on cellular migration of each glycolipid preparation compared to untreated (0) and 1% (*v*/*v*) methanol vehicle control (V. ctrl) on HaCaT cells (**A**), SK-MEL-28 cells (**B**) and a direct comparison of both cell types treated with glycolipids (**C**). Data shown are the areas of cell-free zones measured in a scratch to the cellular monolayer following 24 h of treatment. Measurements were determined from analysis of randomly selected images using Image J software. Statistical significance was determined from three independent experiments via one-way ANOVA followed by Dunnett’s multiple comparison test, * = *p* ≤ 0.05 for the individual cell lines (**A**,**B**) and two-way ANOVA followed by Bonferroni’s multiple comparison test, * = *p* ≤ 0.05 for comparison of the two cell lines (**C**).

To evaluate the effects of glycolipids on in vitro cell migration in both HaCaT and SK-MEL-28 cells, a scratch was made vertically across a monolayer of each cell line. Cells were then treated with each glycolipid preparation at 20 µg mL^−1^ (a concentration previously shown not to affect the viability of each cell line). Following 24 h of treatment, we examined the closure of the artificial wounds by measuring the area of the cell-free zones. HaCaT cell treated with both sophorolipid preparations showed a significant decrease in the cell-free zone after 24 h (lactonic SL *p* ≤ 0.01; acidic SL *p* ≤ 0.001). No significant decrease in cell free zones was observed for HaCaT cells treated with both rhamnolipid preparations and SLES (Figure 4a). SK-MEL-28 cells showed a significant increase in the cell-free zone when treated with all four glycolipid preparations (*p* ≤ 0.05), with no significant difference observed in cells treated with SLES (Figure 4b). When directly comparing both cell lines treated with the glycolipid preparations, we observed both lactonic and acidic sophorolipids to significantly inhibit the migration of SK-MEL-28 cells compared to HaCaT cells treated under the same conditions (lactonic SL *p* ≤ 0.01; acidic SL *p* ≤ 0.001) (Figure 4c). Treatment with both rhamnolipid preparations and SLES showed no significant difference between each cell line with regards to cellular migration (Figure 4c). The most profound effect was with acidic sophorolipids, where the percentage of cell free space in the scratch following 24 h incubation was 71.42% for SK-MEL-28 compared with 0.01% for HaCaT. Moreover, there was no change in cell morphology upon examination, indicating the results from scratch assays were not dependent on a decrease in cell viability at the treatment concentrations used (data not shown).

## 4. Discussion

Melanoma is currently considered as one of the deadliest and most aggressive forms of skin cancer with up to 70% of melanoma incidence associated with direct skin exposure to UV-A and UV-B radiations [35]. Although treatment strategies are currently being improved with the increasing understanding of the pathophysiology of melanomas, there are a significant number of challenges affecting their efficacy [36]. It is therefore a priority to reduce the prevalence of melanomas, enhance the treatment of current incidence and reduce treatment costs [37,38]. Sunscreens have long been recommended for use as one of the primary preventive measures against skin cancers [39,40]. Sunscreens come in the form of lotions, ointments, creams, lip balms, etc., and often contain chemically derived surfactants as emulsification or gelling agents. These surfactants have been reported to be a major cause of skin irritations and allergic reactions to the skin of consumers. Hence, there is a growing demand for biocompatible ingredients in sunscreen formulations that have the physicochemical properties of these synthetic surfactants but also biologically active properties, such as selectively preventing melanoma formation [40,41]. Glycolipids are a widely studied class of microbial biosurfactants and have attracted attention for use in the pharmaceutical and biomedical industries owing to their potential advantages such as compatibility with the human skin, low toxicity, anticancer, wound healing and immunomodulatory effects [16]. Glycolipid bioactivity is highly dependent on the chemical structure of the various congeners present in a sample, and as such information obtained from their chemical characterization and structural analysis is always essential [42,43]. In recent decades, there have been a significant number of reports on the bioactivities of glycolipids. However, many of these reports either utilized poorly characterized, impure preparations or only a single class of glycolipids whose proportion and percentage purity may not have been reported [9,26,44,45,46,47,48,49,50,51]. Therefore, in this study, to investigate the effects of microbial glycolipids with different chemical structures on skin cells, the congeners present in the glycolipid preparations utilized were first rigorously characterized. HPLC has been shown to separate individual congeners within glycolipid mixtures and when coupled with MS, each of these congeners can be separated and identified [9]. Following purification of four separate glycolipid preparations used here, HPLC-MS/ESI analysis showed that the molecular mass of congeners (expressed as relative percentage abundance) corresponding to non-acetylated acidic sophorolipids was 100%; diacetylated lactonic sophorolipids was 89.86%; mono-rhamnolipids was 96%; and di-rhamnolipids was 97%. This level of purity is sufficient to relate any effect observed as being caused by the action of glycolipids belonging to each of these four different structural forms.

Several studies have demonstrated the anticancer properties of sophorolipids and rhamnolipids based on their ability to reduce cell viability and induce apoptosis. However, these studies have often focused on cancers of the esophagus, cervix, colon, lungs, liver, breast, etc. with only a few on skin cancer [19,23,25,29]. Moreover, many of these studies utilized only cancer cell lines, with emphasis on glycolipid cytotoxicity and not their low toxicity effects and compatibility with healthy cell lines [28,45,52]. Notwithstanding, the few studies that utilized healthy cell lines only report them for use as control. A recently published report by Hague et al., 2021 on the reactive oxygen species mediation of necrosis by glycolipids, with emphasis on lung, breast and skin cancers [29], is worthy of note. The authors reported the use of normal human fibroblast cells MRC-5 as a control cell line and these healthy cell lines were only utilized in cell viability assays. There was no further analysis to substantiate the possibility of glycolipids selectively targeting cancer cells for destruction [29]. In an attempt to address the aforementioned challenges, we assessed the effects of purified glycolipid subspecies on cell viability in in vitro spontaneously transformed human keratinocyte HaCaT and human malignant melanocyte SK-MEL-28 cell models using an XTT cell viability assay. In view of the fact that the exact range of concentrations of SLES used in sunscreens are often not reported to the Food and Drug Administration (FDA) in the voluntary industry survey program, the treatment concentrations of SLES utilized in the cell viability assays were varied until observable dose response was achieved, and these effects were compared to the treatment concentrations of the glycolipids. The cell viability assay data presented in this study suggested that glycolipids affected the cell viability of both HaCaT and SK-MEL-28 after 24 h of exposure but that this effect was differentially dependent on both molecular structure and concentration. It is important to state that these cytotoxic effects were significantly more detrimental in SK-MEL-28 than in HaCaT cell lines. However, evidence of this trend in detrimental effects was absent in the same cell lines when treated with the synthetic surfactant SLES at concentrations above 60 μg mL^−1^. The differing effects of each glycolipid structural congener were further brought to prominence using IC_50_ analysis, where we observed a trend of lower IC_50_ values in SK-MEL-28 than in HaCaT cell lines for all glycolipids except for the acidic sophorolipids, whose IC_50_ value could not be determined. More importantly, we observed a significantly lower IC_50_ value for lactonic sophorolipids in SK-Mel-28 cells compared to HaCaT cells. These results suggest that lactonic sophorolipids used at a defined concentration may have the ability to reduce the viability of skin cell melanomas with little or no effect on healthy skin cells.

The difference in the cytotoxic effects demonstrated in this study by each of these glycolipid congeners could be attributed to multiple factors, and as a result may differ from previous studies. These factors include, but are not limited to, chemical structure of the glycolipids, congeners present, the surface, intercellular and intracellular organization of the cell lines under study [52]. For instance, the cytotoxic effects of lactonic sophorolipids are reported to be associated with their degree of acetylation, as well as the length and saturation of hydroxy fatty acids [23,25,50]. In a study on anticancer effects of sophorolipids by Shao et al., 2012, the authors reported that diacetylated sophorolipids exhibited a total inhibition of KYSE109 and KYSE450 esophageal cancer cell lines at 30 μg mL^−1^, but it took monoacetylated sophorolipids a 60 μg mL^−1^ treatment concentrations to achieve the same effects. Moreover, diacetylated lactonic sophorolipids with C18 mono-unsaturated fatty acids exhibited a higher cytotoxicity effect (100% inhibition at 30 μg mL^−1^) than those of di-unsaturated (100% inhibition at 60 μg mL^−1^) and saturated fatty acids (only 20% inhibition at 60 μg mL^−1^). However, for acidic sophorolipids, irrespective of their degree of acetylation and the length and saturation of the hydroxy fatty acid, they had few anticancer effects on the esophageal cancer cells. In a more recent report by Lydon et al., 2017, non-acetylated acidic sophorolipids exhibited no significant cytotoxicity effect on either endothelial (HUVECs) or keratinocyte-derived (HaCaT) cell lines at concentrations above 500 μg mL^−1^ [50]. These differences in chemical structure and congeners of sophorolipid subspecies account for the weaker bioactivity of acidic sophorolipids (predominately C18:1) than lactonic sophorolipids (predominantly R1 + R2 = Ac, C18:1), which are all in agreement with this present study. In the case of rhamnolipids, there is a huge variation in the report of their cytotoxicity from one study to the other. While some authors report that di-rhamnolipids have higher cytotoxic effects than mono-rhamnolipids, others report the contrary [27,52,53]. For instance, in a report on the anticancer effects of rhamnolipids produced by *Pseudomonas aeruginosa* on the MCF-7 human breast cancer cell line, the authors demonstrated that mono-rhamnolipids (predominantly Rha-C_10_-C_10_) exhibited higher cytotoxic effects (IC50 = 25.87 μg mL^−1^) on the cancer cell line than di-rhamnolipids (predominantly Rha-Rha-C_10_-C_10_) (IC50 = 31 μg mL^−1^) [52]. On the contrary, another report on anticancer effects of rhamnolipids on the aforementioned breast cancer cell line revealed that di-rhamnolipids (predominantly Rha-Rha-C_10_-C_10_) expressed higher cytotoxic effects than mono-rhamnolipids (predominantly Rha-C_10_-C_10_) with minimum inhibitory concentrations of 1 and 5 μg mL^−1^, respectively [53]. In agreement with the latter studies, a potentially determining factor for the cytotoxic effects of rhamnolipids we observed could emanate from the interaction between the specific chemical structure of the rhamnolipid subspecies and the membrane biophysical properties of a particular cell type [52]. Considering that the functional groups on cancer cell membrane surfaces are more negatively charged than healthy cells, we suggest that the higher cytotoxic effects we observed by di-rhamnolipids, particularly in SK-MEL-28 in this present study could be attributed to the interaction between the more negatively charged functional groups on melanoma cell surfaces and the less anionic di-rhamnolipids. Moreover, beyond the seeming contradictions over the cytotoxic effects of rhamnolipids demonstrated in several studies, the purity of rhamnolipids used in this present study has been clearly defined, thus providing a good foundation for further future studies when it comes to understanding the underlying mechanisms influencing rhamnolipid cytotoxic effects.

Morphological examination post glycolipid treatment at high concentration, followed by AO and PI staining, suggest that the pattern of cell death induced was necrosis. Necrotic cell death was again dependent on both glycolipid congeners and concentration with di-rhamnolipids, lactonic sophorolipids and SLES showing an effect at 100 μg mL^−1^ and the remaining glycolipids showing an effect at 500 μg mL^−1^_._ Necrosis induction by surfactants is hypothesized to be associated with surfactant intercalation into the lipid membrane of cells, thereby causing a change in carbon chain rearrangement of the cell membranes. Further increasing glycolipids concentration may result in the dehydration of the phospholipid bilayer, which may in turn affect cellular adhesion, membrane functionality and ultimately cause cell death [26]. The consistency of the morphological observations and the cell viability results are indications that lower concentrations of glycolipids would be required to have detrimental effects on malignant melanocytes than on healthy skin cells, hence fitting them for potential use in novel anticancer therapy. However, it must be noted that although glycolipids had relatively few effects on keratinocytes, the demonstration of necrosis as glycolipid-associated cell death in the present study suggest that glycolipids could affect healthy cells in vivo. Therefore, further in vitro and in vivo studies supported by flow cytometry-based assays, targeted at investigating a gentler mechanism of glycolipid-associated cell death such as apoptosis, will be essential to validate the anticancer effects of glycolipids.

The ability of cancerous cells to invade the dermis or elevate the epidermis to form nodules is a critical event in melanoma development. At this stage, lesions may metastasize to other organs of the body. Metastasized melanomas account for the main cause of death in melanoma patients. The majority of current targeted melanoma therapies have not been successful [54]. In light of this challenge, we further assessed the properties of glycolipids by studying whether glycolipids could inhibit the migration of SK-MEL-28 after an artificial wound was made across a monolayer of cells. Analysis of cell-free zones revealed that, when compared to the untreated control, both acidic and lactonic sophorolipids significantly inhibited the migration of melanoma cells after 24 h. In a similar report by Ribeiro et al., 2013, the authors demonstrated treatment with diacetylated lactonic sophorolipids (C18:1) inhibited the migration of the breast cancer cell line, MDA-MB-231 by 41.5% after 24 h [24]. Tumor metastasis is mostly initiated by the invasion of the basement membrane of surrounding tissue by cells migrating through extracellular matrixes [55]. Therefore, targeting factors such as the expression of extracellular matrix proteins (e.g., metalloproteinases), upregulation and stiffness of the extracellular matrix are important in the development of antimetastatic therapies [55,56,57]. Inhibition of matrix metalloproteinase expression in mouse metastatic melanoma cell line (B16-F10) by Hinokitiol, a natural bioactive compound, has been reported [56]. More recently, Hague et al., 2021 also reported the inhibitory effect of lactonic sophorolipids on migration of skin melanoma, lung and breast cancers as a consequence of disrupting the existing network of actin filaments [29]. Although the antimetastatic mechanisms of glycolipids have not been widely explored and as a result these mechanisms are not currently understood, the evidence of their migration inhibitory effects provided in this current study are important for future research.

## 5. Conclusions

This study shows that microbial glycolipids affect skin cells in a differential manner dependent on their chemical structure and have a greater detrimental effect on malignant melanoma cells compared to healthy human keratinocytes. Additionally, our findings suggest that glycolipids prevented the migration of a melanoma cell line, therefore they have antimetastatic potential. As a less toxic surface-active agent with added anticancer biological activity, glycolipids could form a substitute to SLES in commercially available sunscreens. Although the specific mechanisms are not fully understood, with these findings and further future research using appropriate in vivo or ex vivo models, we may be on the road to finding novel anticancer therapy with the potential to selectively target malignant melanomas for destruction.

## Figures and Tables

**Table 1:** HPLC-MS analysis showing the molecular structure and relative percentage abundance of the congeners comprising each glycolipid preparation utilized in this study. (* Denotes contaminant congeners).

	RT (min)	*m*/*z* Value	Compound	Mw (Da)	Relative (%)
**Acidic-SL Preparation**	13.03	595.3	Acidic, C16:0	596	6.31
	13.64	619.1	Acidic, C18:2	620	0.72
	13.88	621.3	Acidic, C18:1	622	65.53
	14.46	623.3	Acidic, C18:0	624	14.93
	7.28	637.3	Acidic, R1 = Ac, C16:0	638	3.58
	15.21	665.3	Acidic, 1Ac, C18:0	666	1.14
	15.01	663.2	Acidic, R1 = Ac, C18:1	664	2.55
	15.62	679.2	Acidic, R1 + R2 = Ac, C16:0	680	0.56
	16.19	705.2	Acidic, R1 + R2 = Ac, C18:1	706	2.90
	16.95	707.3	Acidic, R1 + R2 = Ac, C18:0	708	0.85
	13.03	721.2	Acidic, R1 = Ac, C22:0	722	0.65
	15.57	791.3	Acidic, R1 + R2 = Ac, C24:0	792	0.29
**Lactonic-SL Preparation**	12.86	705.1	Acidic, R1 + R2 = Ac, C18:1 *	706	10.14
	14.21	645.1	Lactonic, R1 = Ac, C18:1	646	3.24
	16.36	685.1	Lactonic, R1 + R2 = Ac, C18:2	686	15.95
	17.36	687.1	Lactonic, R1 + R2 = Ac, C18:1	688	63.40
	18.93	689.1	Lactonic, R1 + R2 = Ac, C18:0	690	7.27
**Mono-RL Preparation**	19.62	332.9	Rha-C_10_	334	1.35
	19.65	502.9	Rha-C_10_-C_10_	504	84.40
	19.60	505.0	Rha-Rha-C_12:1_ *	506	2.97
	21.77	528.9	Rha-C_10_-C_12:1_/ Rha-C_12:1_-C_10_	530	6.63
	23.12	530.9	Rha-C_10_-C_12_/ Rha-C_12_-C_10_	532	4.65
**Di-RL Preparation**	11.03	332.9	Rha-C_10_ *	334	0.19
	9.78	479.0	Rha-Rha-C_10_	480	23.84
	29.81	502.9	Rha-C_10_-C_10_ *	504	0.59
	29.79	505.0	Rha-Rha-C_12:1_	506	0.15
	12.96	507.0	Rha-Rha-C_12_	508	1.15
	31.36	528.0	Rha-C_10_-C_12:1_/Rha-C_12:1_-C_10_ *	530	1.04
	31.33	530.9	Rha-C_10_-C_12_/Rha-C_12_-C_10_ *	532	0.27
	15.36	621.0	Rha-Rha-C_10_-C_8_/Rha-Rha-C_8_-C_10_	662	0.95
	16.29	647.1	Rha-Rha-C_10_-C_10:1_/Rha-Rha-C_8_-C_12:1_	648	0.26
	17.30	649.1	Rha-Rha-C_10_-C_10_	650	57.99
	17.88	663.0	Rha-Rha-C_10_-C_10_-CH_3_	664	0.27
	18.15	675.1	Rha-Rha-C_10_-C_12:1_/Rha-Rha-C_12:1_-C_10_	676	4.18
	19.83	677.1	Rha-Rha-C_10_-C_12_/Rha-Rha-C_12_-C_10_	678	8.72
	21.48	703.1	Rha-Rha-C_10_-C_14:1_/Rha-Rha-C_12:1_-C_12_	704	0.18
	23.00	705.0	Rha-Rha-C_12_-C_12_	706	0.13
	31.18	988.0	Rha-Rha-C_14_-C_14_-C_14_	989	0.07

**Table 2:** Mean IC_50_ values (± SEM) of each glycolipid preparation for both HaCaT and SK-Mel-28 cell lines from three independent experiments. ND: not determined. Statistical significance was determined using an unpaired *t*-test (*p* = ≤ 0.05).

Surfactant	IC_50_ (± SEM) (μg mL^−1^)		Significant	*p* Value
	HaCaT	SK-Mel-28		
Lactonic sophorolipid (0–100 μg mL^−1^)	62.62 (1.34)	53.83 (1.64)	Yes	0.0142
Acidic sophorolipid (0–100 μg mL^−1^)	ND	ND	-	-
Di–rhamnolipid (0–100 μg mL^−1^)	47.57 (2.76)	40.79 (0.85)	No	0.0789
Mono–rhamnolipid (0–100 μg mL^−1^)	ND	ND	-	-
SLES (0–100 μg mL^−1^)	65.50 (1.26)	65.06 (0.46)	No	0.7571
Acidic sophorolipid (0–500 μg mL^−1^)	ND	ND	-	-
Mono-rhamnolipid (0–500 μg mL^−1^)	628.3 (47.61)	570.4 (44.11)	No	0.4228

## Data Availability

The data presented in this study are available upon request from the corresponding author.

## Supplementary Materials

The following are available online at https://www.mdpi.com/article/10.3390/pharmaceutics14020360/s1: Figure S1: HPLC-MS profile of each glycolipid preparation utilized in this study, (acid sophorolipid, lactonic sophorolipid, mono-rhamnolipid and di-rhamnolipid), with predominant peaks identified; Figure S2: Morphological assessment of the pattern of cell death induced by glycolipids in HaCaT and SK-MEL-28 cells using AO/PI staining following a 24 h treatment with SLES at 100 μg mL^−1^.
