# Supplementary material for: Biosurfactants as Anticancer Agents: Glycolipids Affect Skin Cells in a Differential Manner Dependent on Chemical Structure
Source: Pharmaceutics. 2022 Feb 4;14(2):360. doi: 10.3390/pharmaceutics14020360 (PMC8874633; doi:10.3390/pharmaceutics14020360)
Supplement: Supplementary file 1 [file pharmaceutics-14-00360-s001.zip › pharmaceutics-1579249-sup.pdf]

pharmaceutics

MDPI

Supplementary Materials: Biosurfactants as Anti-cancer Agents: Glycolipids Affect Skin Cells in a Differential Manner Dependent on Chemical Structure

Simms A. Adu, Matthew S. Twigg, Patrick J. Naughton, Roger Marchant, and Ibrahim M. Banat

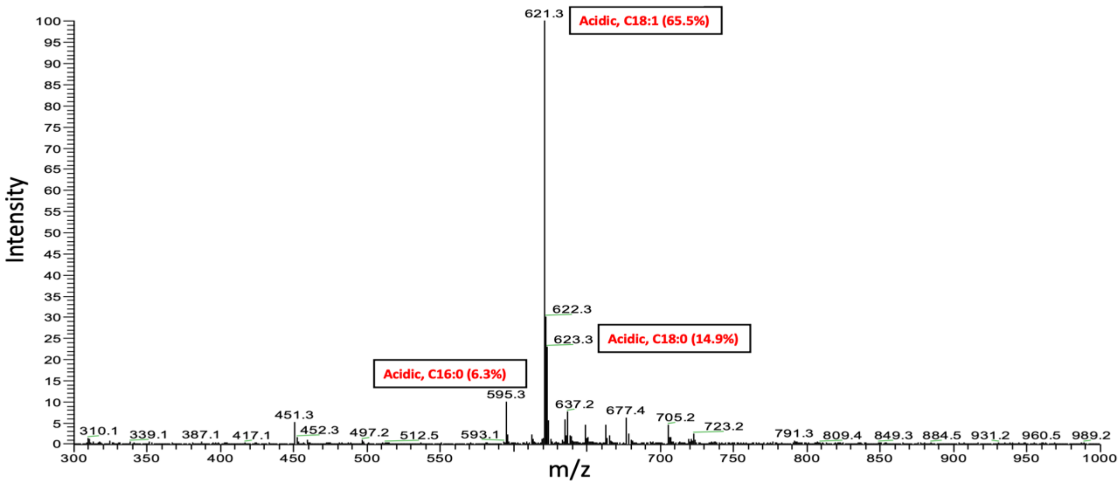

Acidic SL: LC-MS/ESI mass spectrum in negative ion mode

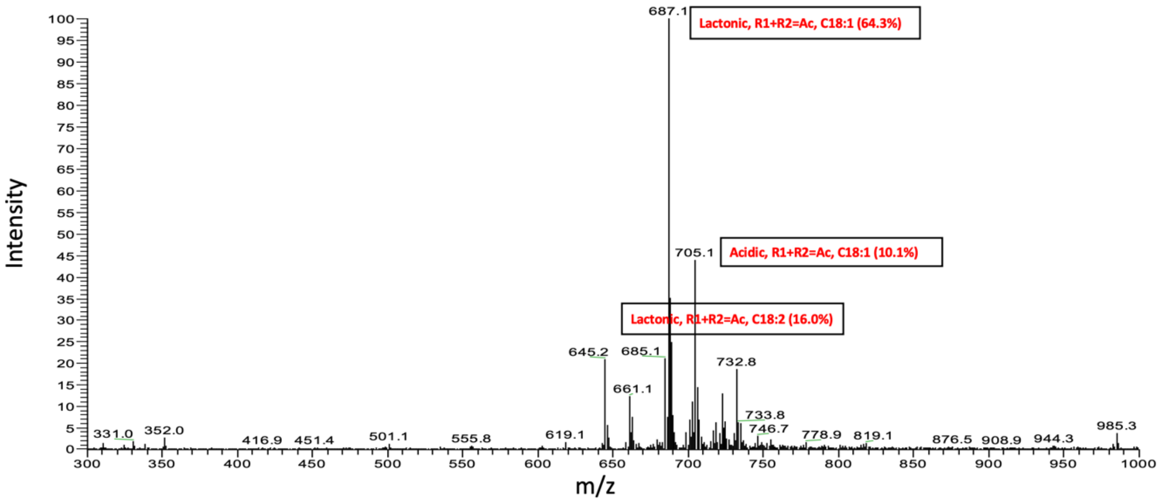

Lactonic SL: LC-MS/ESI mass spectrum in negative ion mode

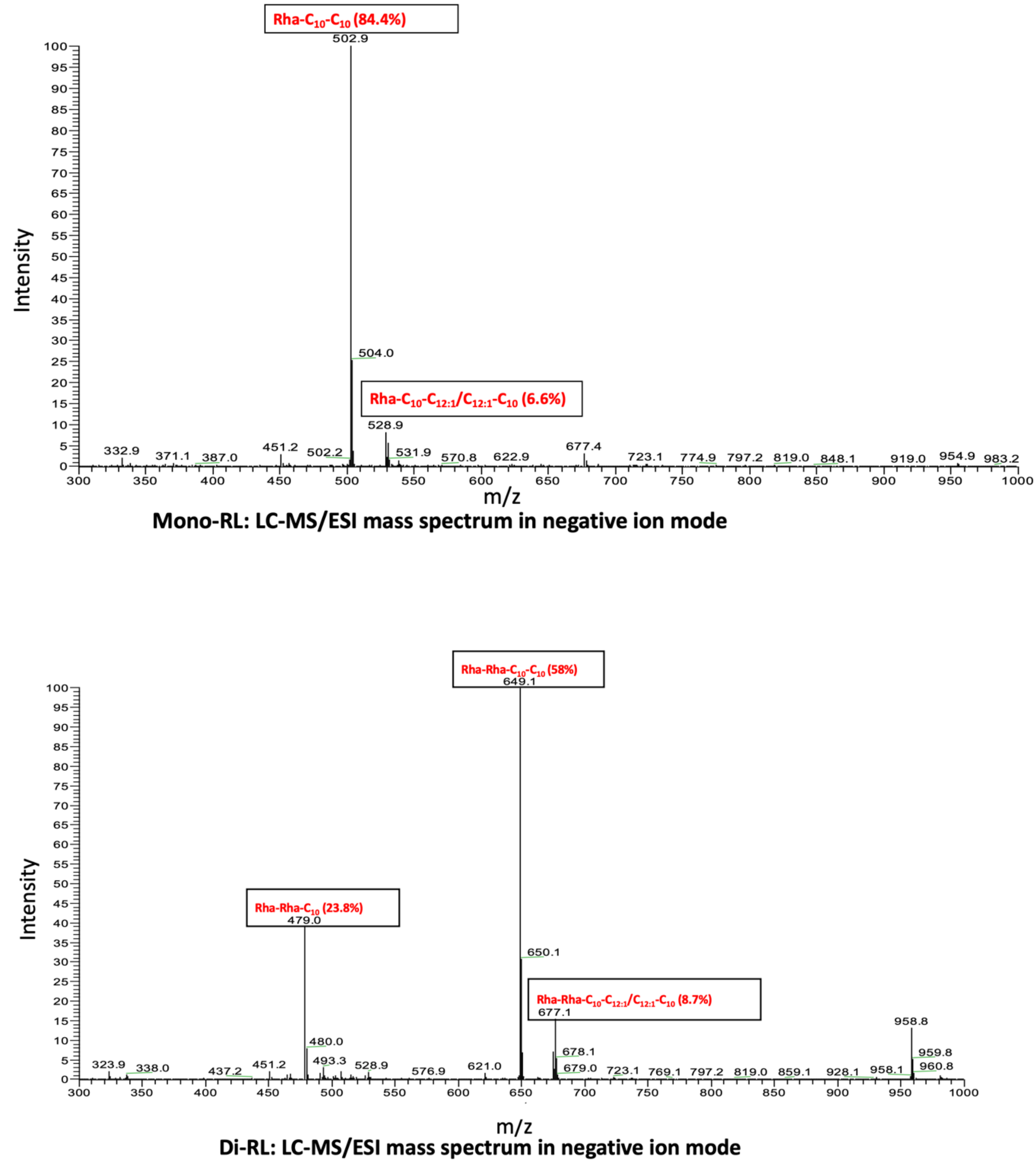

Figure S1. HPLC-MS profile of each glycolipid preparation utilized in this study, (acidic sophorolipid, lactonic sophorolipid, mono-rhamnolipid and di-rhamnolipid), with predominant peaks identified.

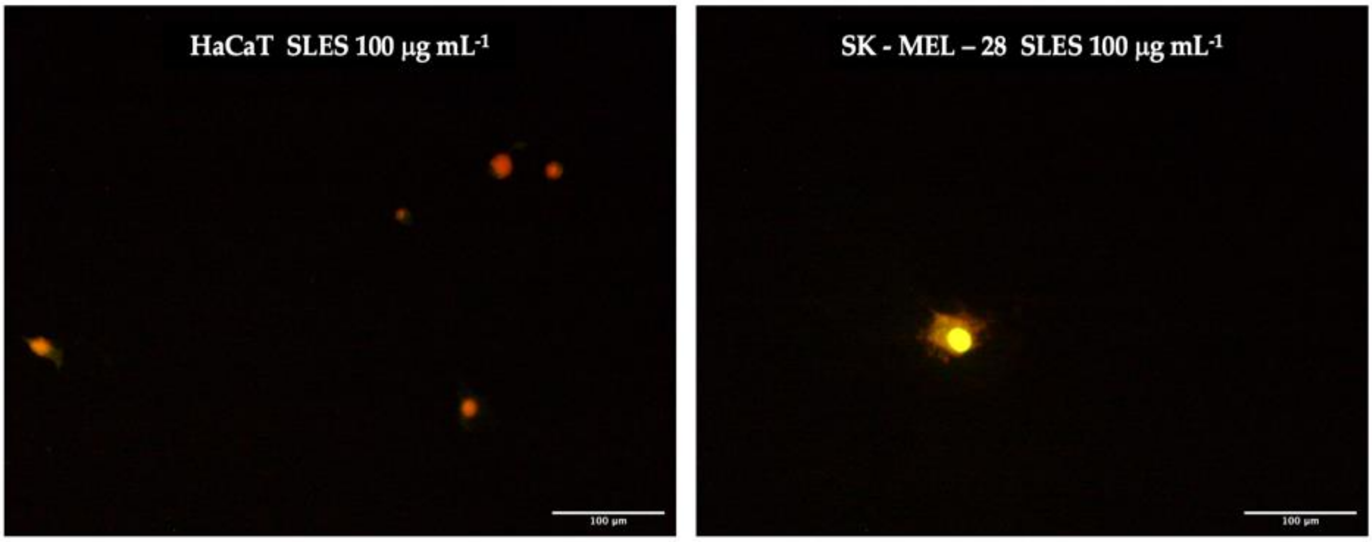

Figure S2. Morphological assessment of the pattern of cell death induced by glycolipids in HaCaT and SK-MEL- 28 cells using AO/PI staining following a 24 h treatment with SLES at 100 μg mL^{-1}
